# Effects of Vitamin D_3_ and Paricalcitol on Immature Cardiomyocytes: A Novel Role for Vitamin D Analogs in the Prevention of Cardiovascular Diseases

**DOI:** 10.3390/nu5062076

**Published:** 2013-06-07

**Authors:** Stefania Pacini, Gabriele Morucci, Jacopo J. V. Branca, Stefano Aterini, Marcello Amato, Massimo Gulisano, Marco Ruggiero

**Affiliations:** 1 Department of Experimental and Clinical Medicine, University of Firenze, 50134 Firenze, Italy; E-Mails: stefania.pacini@unifi.it (S.P.); gabriele.morucci@unifi.it (G.M.); jacopo.branca@libero.it (J.J.V.B.); massimo.gulisano@unifi.it (M.G.); 2 Division of Nephrology and Hemodialysis, Prato Hospital, 59100 Prato, Italy; E-Mails: saterini@usl4.toscana.it (S.A.); mamato@usl4.toscana.it (M.A.); 3 Department of Biomedical, Experimental and Clinical Sciences, University of Firenze, 50134 Firenze, Italy

**Keywords:** vitamin D_3_, paricalcitol, GcMAF, cardiomyocytes, kidney disease

## Abstract

Cardiovascular diseases are more prevalent in patients with chronic kidney disease than in the general population and they are considered the leading cause of death in patients with end-stage renal disease. The discovery that vitamin D_3_ plays a considerable role in cardiovascular protection has led, in recent years, to an increase in the administration of therapies based on the use of this molecule; nevertheless, several studies warned that an excess of vitamin D_3_ may increase the risk of hypercalcemia and vascular calcifications. In this study we evaluated the effects of vitamin D_3_, and of its selective analog paricalcitol, on immature cardiomyocytes. Results show that vitamin D_3_ induces cAMP-mediated cell proliferation and significant intracellular calcification. Paricalcitol, however, induces cell differentiation, morphological modifications in cell shape and size, and no intracellular calcification. Furthermore, vitamin D_3_ and paricalcitol differently affect cardiomyoblasts responses to acetylcholine treatment. In conclusion, our results demonstrate that the effects of vitamin D_3_ and paricalcitol on cardiomyoblasts are different and, if these *in vitro* observations could be extrapolated *in vivo*, they suggest that paricalcitol has the potential for cardiovascular protection without the risk of inducing intracellular calcification.

## 1. Introduction

The National Kidney Foundation Task Force on Cardiovascular Disease recommends that chronic kidney disease (CKD) patients be considered among the highest risk group for developing cardiovascular (CV) disease [1]. In fact, CV disease is more prevalent in patients with CKD than in the general population [2] and it is the leading cause of death in patients with end-stage renal disease [1]. It has been reported that nearly 30% of deaths among patients with CKD are to be attributed to cardiovascular causes [3].

CKD associated hyperparathyroidism and mineral metabolism disorders, such as hyperphosphatemia, have been significantly correlated with vascular and visceral calcifications, and consequently with increased risk of CV disease [4]. 

In addition to vascular problems, left ventricular hypertrophy, subclinical systolic dysfunction, and diastolic dysfunction have been consistently observed in subjects with CKD [5]. It is conjectured that abnormal diastolic function is an independent predictor of decreased aerobic capacity during the early stages of CKD [6]. 

Nevertheless, even though numerous factors have been implicated in the aetiology of the cardiac abnormalities observed in patients with CKD, the exact aetiology of the observed cardiac changes still remains unclear. A number of cardiac pathologies, including heart failure, are associated with alterations in myocardial energy metabolism [7] as well as with the activation of different intracellular signal transduction pathways. 

Vitamin D_3_ (in its active form, *i.e.*, calcitriol or 1,25-dihydroxyvitamin D_3_), as many other direct-acting positive inotropic agents, stimulates cyclic AMP (cAMP) formation [8], a major mediator of the amplitude and time course of cardiac contraction [8]. This cAMP-mediated inotropic effect occurs through activation of a variety of cAMP-dependent protein kinases that are capable of phosphorylating a series of proteins that affect the energetic status of the cells and alter the flux of calcium in the sarcoplasm [8].

In fact, calcium release and energetic systems are strictly and subtly modulated by different molecules, and an imbalance of sympathetic and parasympathetic drive to the heart represents an important risk factor for cardiac death in patients with renal insufficiency [9]. 

Since vitamin D_3_, together with dietary restrictions and phosphate binders, represents the primary medication to treat secondary hyperparathyroidism and the associated calcium and phosphate metabolic alterations in CKD, interest in the role of vitamin D_3_ axis in the cardiovascular system recently increased. Thus, the vitamin D axis, which plays a critical role in the development of CKD, includes vitamin D, the polymorphic vitamin D receptor (VDR), and the vitamin D-binding protein (Gc-globulin) [10]. That is the precursor of a potent macrophage activating factor (GcMAF), which has potent effects on the immune system and angiogenesis [11]. 

In parallel, with this interest for beneficial effects [12], however, other studies warned that an excess of vitamin D_3_ increases the risk of hypercalcemia and vascular calcifications thus increasing the risk for CV and reducing survival in patients with CKD [13,14].

In this study we investigated the effect of vitamin D_3_ as well as of one of its non-hypercalcemic analogs, paricalcitol, on cultured cardiomyocytes. Paricalcitol (19-nor-1,25-dihydroxyvitamin D_2_) is a vitamin D_3_ analog, acting as a selective VDR activator; for this reason it might provide a vitamin D_3_-like protective efficacy without the hypercalcemic and hypophosphatemic side effects of vitamin D_3_, thus representing a potentially useful tool against the cardio-renal syndrome [12,15]. In particular, to better evaluate the modifications of the intracellular energy status as well as the modifications in signal transduction, in this study we compared the effects of vitamin D_3_, to those of paricalcitol in a myoblastic cell line H9c2, not completely differentiated showing electrophysiological and biochemical properties of cardiac muscle tissue [16]. Proliferation, mitochondrial activity, morphological alterations, calcification, cAMP pathway activation, and response to acetylcholine were thus investigated.

## 2. Experimental Section

### 2.1. Pharmacological Agents

Vitamin D_3_ and Paricalcitol were respectively obtained from Sigma Aldrich, Milano, Italy and from Abbott, Roma, Italy. Due to differences in test compounds, two different treatment vehicles were used during the study: 100% ethanol (for vitamin D_3_) and 30% polyethylene glycol/20% ethanol in water (for paricalcitol). Preliminary experiments did not show significant differences among different concentrations of vehicles in cell responses (not shown). Therefore, the different vehicle concentrations data were pooled for the analysis in this report. Vitamin D binding protein-derived macrophage activating factor (GcMAF) was obtained from Immuno Biotech Ltd. (Guernsey, Channel Islands). β-Glycerol-phosphate (β-GP), ethanol, polyethylene glycol, and acetylcholine were obtained from Sigma Aldrich, Milano, Italy. 

### 2.2. Cell Cultures

H9c2 cells, immortalized ventricular myocytes derived from rat (*Rattus norvegicus*) embryos, were obtained from the “Istituto Zooprofilattico della Lombardia e dell’Emilia Romagna”, Brescia, Italy. H9c2 is a myoblast cell line not yet completely differentiated into non-proliferating myocytes/myotubes and with electrophysiological and biochemical properties of both skeletal and cardiac muscle tissue. Cell were grown in a monolayer culture at 37 °C in a 5% CO_2_ humidified atmosphere in Dulbecco’s modified eagle’s medium (DMEM) supplemented with 10% foetal bovine serum (FBS). The medium was renewed every two to three days, when the cells reached sub-confluence (70%–80%). Vitamin D_3_ and paricalcitol were added to the cells at the following concentrations: [1 nM], [10 nM], [100 nM], and [300 nM]. Treatment with acetylcholine was performed with and without vitamin D_3_ and paricalcitol [300 nM] at the concentration of [10 μM] for 1 h. 

### 2.3. Cell Viability and Proliferation

The effects of vitamin D_3_ and paricalcitol on H9c2 cell lines, were evaluated by the cell viability assay using the 2-(2-methoxy-4-nitrophenyl)-3-(4-nitrophenyl)-5-(2,4-disulf-ophenyl)-2*H* tetrazolium monosodium salt (WST-8) reagent (Sigma Aldrich, Milano, Italy), according to the manufacturer’s protocol. This test is based on a colorimetric conversion of a tetrazolium salt (WST-8) to water-soluble formazan products as previously described [17]. H9c2 cells were plated at density of 2 × 10^4^ per well in a 96-well plate with fresh growth medium for one day. Then, vitamin D_3_ and paricalcitol were diluted to appropriate concentration and added to the culture medium for 48 h. After the exposure-time, WST-8 solution was added to each well and the micro-plate incubated for 4 h at 37 °C. Cell viability was measured by a micro-plate reader (*Multiscan FC/FL6111900 model*, Thermo Fisher Scientific, M-Medical, Milano, Italy) at 450 nm after incubation-time. To directly evaluate cell proliferation, a cell count by a haemocytometer (Housser Scientific, Horsham, PA, USA) was also performed. Also the evaluation of cardiomyocytes response to acetylcholine in the presence of vitamin D_3_ and paricalcitol was evaluated by cell viability assay. In each experiment, at least seven wells were used for each experimental point.

### 2.4. Cell Morphology

To evaluate changes in H9c2 cell morphology before and after vitamin D_3_ and paricalcitol exposure, contrast phase microscopy and Haematoxylin-Eosin staining were performed.

Briefly, H9c2 cells were seeded on a cover slip at the density of 10 × 10^4^ cells/cover slip.

Cells were grown in culture medium with different concentrations of vitamin D_3_ and paricalcitol for 48 h. For the Haematoxylin-Eosin staining, H9c2 cells were fixed with paraformaldehyde 0.5% in [0.1 M] PBS (phosphate buffer saline) and dried for 2 h. Then, cells were stained with Haematoxylin-Eosin dye following a standard protocol.

Cell morphology was evaluated by optical microscope (XDS-2 + M-795 model, Optika Microscope, Milano, Italy) and digital images were captured.

After the treatment at different concentration of vitamin D_3_ and paricalcitol for 48 h, cell size of H9c2 was measured by Adobe Pohotoshop CS2 software (9.0 version, Adobe System Incorporated 1990–2005; Adobe Systems Inc., San Jose, CA, USA) and Scion Image software (Beta 3b freeware version; based on NIH Image for Macintosh by Wayne Rasband, National Institute of Health, USA and modified for Windows by Scion Corporation; July, the 23rd 1998; Scion Co., Frederick, MD, USA). Images of H9c2 in Haematoxylin-Eosin staining were captured using a digital camera (DIGI-full HD video/photo camera, Optika Microscopes, Milano, Italy). Digital images were adjusted by Adobe Photoshop CS2 to remove from the picture everything (background) except the cells and then H9c2 cells size was measured by Scion Image.

### 2.5. cAMP Assay

H9c2 cells were cultured at density of 1 × 10^4^ cells per well in a 6-well plate with DMEM supplemented with 10% FBS and exposed at [1 nM], [10 nM], [100 nM], and [300 nM] vitamin D_3_ and paricalcitol for 48 h. The cAMP intracellular level was evaluated by a direct competitive immunoassay for sensitive and quantitative determination using a cAMP assay kit (Abnova, Heidelberg, Germany) according to the Manufacturer’s instructions. After the treatments, the experiment was blocked adding [0.1 M] HCl. Then, after scraping cells from the substrate, the suspension was centrifuged for 10 min at 2340 rpm. The supernatant was assayed for cAMP concentration by a micro-plate reader (Multiscan FC/FL6111900 model, Thermo Fisher Scientific, M-Medical, Milano, Italy) at 450 nm after incubation-time. cAMP values were normalized to the assayed proteins.

### 2.6. Analysis of H9c2 Calcification

To evaluate the deposits of calcium in H9c2 after vitamin D_3_ and Paricalcitol exposure, cells were seeded on cover slip at density 10 × 10^4^ per cover slip and cultured in their culture medium (DMEM + 10% FBS). The medium was substituted with fresh culture medium every two days. After five days from confluence, fresh medium containing different concentration of vitamin D_3_ and paricalcitol was used for a further seven days. To amplify the calcification effects of vitamin D_3_ and paricalcitol, the medium was enriched with [4 mM] β-GP (calcification media). Quantification of calcium deposits was performed by von Kossa staining (von Kossa Kit, Bio Optica, Milano, Italy); cells were soaked in a lithium carbonate solution (for 10 min) to prevent false results caused by the presence of uric acid and urates in the samples; then, cells were treated with a silver nitrate solution, left in dark for 1 h, and then a reduction solution was applied for 5 min; then, samples were treated with a sodium sulphite solution for 5 min and with a Mayer’s Carmalum solution to contrast the calcium deposits. The intracellular calcification was evaluated by optical microscope (XDS-2 + M-795 model, Optika Microscope, Milano, Italy) and images of H9c2 cell line in von Kossa staining were captured using a digital camera (DIGI-full HD video/photo camera, Optika Microscopes, Milano, Italy) and then analyzed.

### 2.7. Statistics

All values are means ± standard error (SE) for at least three determinations. Differences between experimental points were evaluated by Student’s *t*-test. *p* was considered statistically significant when *p* < 0.05.

## 3. Results

### 3.1. Cell Viability and Proliferation

**Figure 1:**
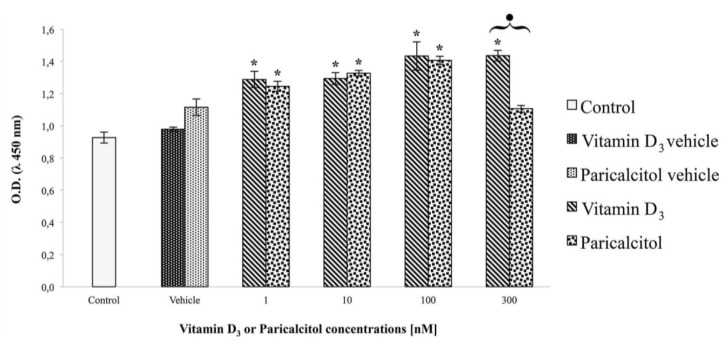
Cell viability assay in H9c2 cells shows that [1 nM], [10 nM], and [100 nM] Vitamin D_3_ and Paricalcitol induce no significant differences in cell viability in comparison to control cells (both untreated and treated with appropriate vehicles). On the contrary, a significant difference (*p* < 0.05) is observed when H9c2 are treated with [300 nM] vitamin D_3_. Data are expressed as means ± S.E.M. (standard error of the mean) *vs.* control. * Statistically significant difference (*p* < 0.05) of vitamin D_3_ (or paricalcitol) *vs.* controls (untreated cells and vehicles). Black dot indicates a statistically significant difference between vitamin D_3_ and paricalcitol.

Data obtained from cell viability assay show that treatment for 48 h with vitamin D_3_ or paricalcitol induces a similar dose-dependent response. In particular, [1 nM], [10 nM], and [100 nM] vitamin D_3_ and paricalcitol induce a statistically significant increase in cell viability in comparison to untreated control cells, as well as in comparison to cells treated with appropriate vehicles alone (Figure 1). 

However, when cells are treated with [300 nM] vitamin D_3_ or [300 nM] paricalcitol a significant difference (*p* < 0.05) in cell viability occurs (Figure 1); vitamin D_3_ induces an increase in cell viability while paricalcitol induces a significant decrease.

**Figure 2:**
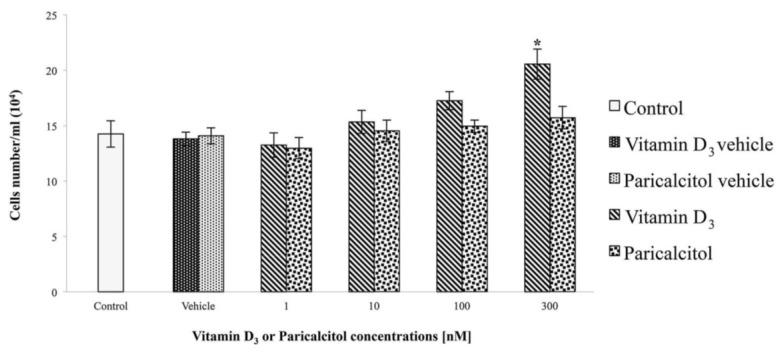
A significant increase in cell density is observed when cells are treated with [300 nM] Vitamin D_3_ in comparison to controls. Data are expressed as means ± S.E.M. *vs.* control. * Statistically significant difference (*p* < 0.05) of vitamin D_3_ *vs.* controls (untreated cells and vehicles).

Since cell viability is correlated, but not completely superimposable to cell proliferation, we tested the effects of vitamin D_3_ and paricalcitol on cell proliferation by direct cell count. Results show that only when cells are treated with [300 nM] vitamin D_3_ a significant increase in cell number and density occurs (Figure 2). No significant increases in cell number and density are observed comparing controls (untreated or treated with appropriate vehicles) to exposed cells with all the other experimental concentrations and stimuli (Figure 2).

### 3.2. cAMP Levels

**Figure 3:**
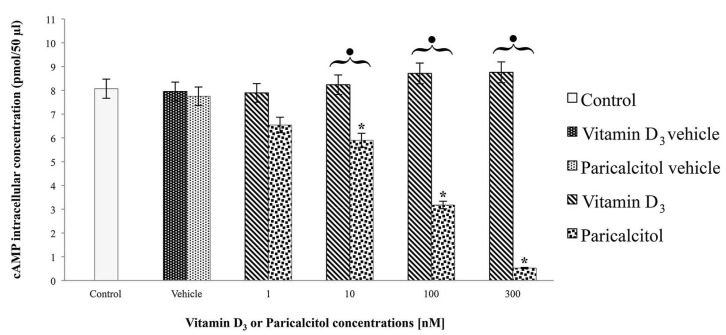
A different pattern in cAMP levels can be observed when cells are treated with vitamin D_3_ or Paricalcitol. Data are expressed as means ± S.E.M. *vs.* control. * Statistically significant difference (*p* < 0.05) of vitamin D_3_ (or paricalcitol) *vs.* controls (untreated cells and vehicles). Black dots indicate statistically significant differences between vitamin D_3_ and paricalcitol.

Data obtained after treatment of H9c2 cells for 48 h, with vitamin D_3_ and paricalcitol at different concentrations, show different trends for the two molecules: vitamin D_3_ induces a dose-dependent increase in cAMP level (even though not statistically significant), while paricalcitol induces a dose-dependent decreases in comparison to controls (Figure 3). For [10 nM], [100 nM], and [300 nM] paricalcitol, differences in cAMP level are statistically significant in comparison to controls. At the same concentrations the differences in cAMP levels induced by treatment with vitamin D_3_ and paricalcitol are also statistically significant.

### 3.3. Cell Morphology

**Figure 4:**
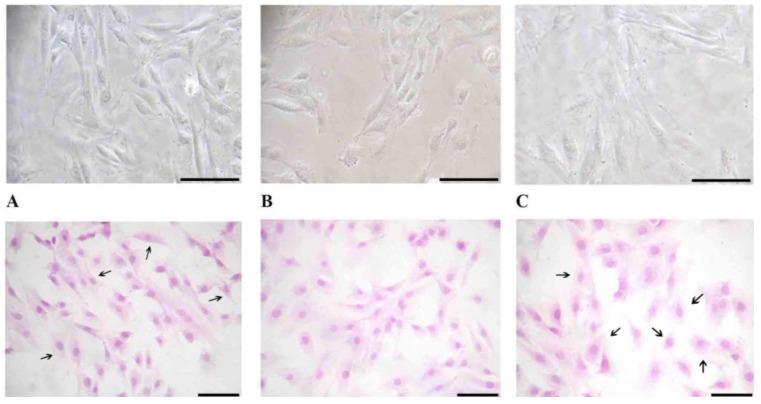
H9c2 cell morphology analysis by contrast phase microscopy and after Haematoxylin-Eosin staining. Panels **A**: untreated and vehicles treated cells observed by contrast phase microscopy (upper panel) and after Haematoxylin-Eosin staining (lower panel). Cells are spindle-like and with evidently thin and numerous cytoplasmic elongations (arrows). Panels **B**: cells treated with [300 nM] vitamin D_3_. Cells do not show significant morphological changes in comparison to controls both when observed by contrast phase microscopy (upper panel) and after staining (lower panel). Panels **C**: cells treated with [300 nM] paricalcitol in some cases appear roundish and with a reduced number of cytoplasmic elongations (arrows). Upper panels: total magnification 150×. Lower panels: total magnification 100×. Bars: 100 μm.

Significant changes in cell morphology are observed when cell are treated with [300 nM] paricalcitol. No significant changes are observed when cells are treated both with lower concentrations of paricalcitol, and with vitamin D_3_. Direct observation by contrast light microscopy, as well as after Haematoxilyn-Eosin staining, reveals relevant modifications in cell shape between treated and control cells (Figure 4). Control cells show a spindle-like shape with an oval nucleus, localized in the central part of the cell, and several elongations arising from the cell body. Cells tend to establish contact with each other so that cytoplasmic elongations appear sometimes very long and thin (Figure 4A).

**Figure 5:**
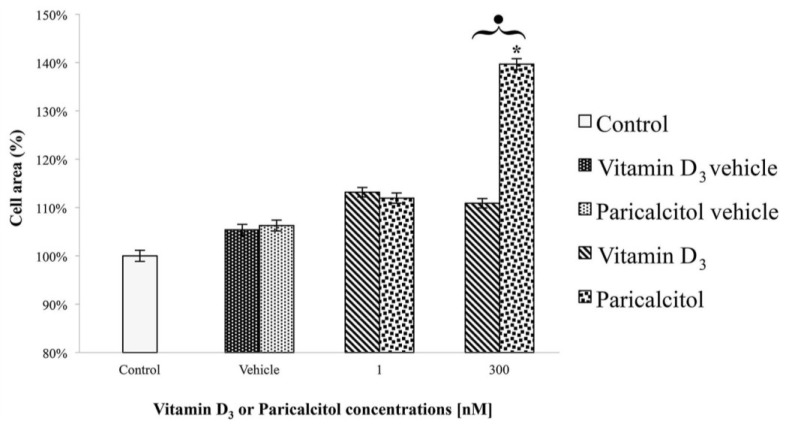
A significant difference in cell size is observed when cells are treated with [300 nM] paricalcitol. Data are expressed as means ± S.E.M. *vs.* control. * Statistically significant difference (*p* < 0.05) of paricalcitol *vs.* controls (untreated cells and vehicles). Black dot indicates a statistically significant difference between vitamin D_3_ and paricalcitol.

Treatment of H9c2 cells with [300 nM] vitamin D_3_ does not induce significant changes in cell morphology (Figure 4B), whereas the same concentration of paricalcitol induce significant modification (Figure 4C): a great number of cells appear roundish instead of spindle-like and the number of cytoplasmic elongations appears greatly reduced. The changes in cell morphology are associated with an increase in cell size. In fact, when cells are treated with [300 nM] paricalcitol but not with the same concentration of vitamin D_3_ a significant difference in cell size can be observed (Figure 5). After treatment with [300 nM] paricalcitol, cell diameter increases from 15 ± 1.2 μm (mean ± S.E.M.) to 20 ± 1.0 μm (mean ± S.E.M.). Cell size, expressed as cell area and calculated by appropriate software as described in the Section 2 Materials and Methods, appears about 40% wider: after the treatment with [300 nM] paricalcitol cell surface increases from 106.96 ± 7.36 μm^2^ (mean area ± SEM) to 211.16 ± 6.12 μm^2^ (mean area ± S.E.M.).

### 3.4. Acetylcholine and Cell Viability

**Figure 6:**
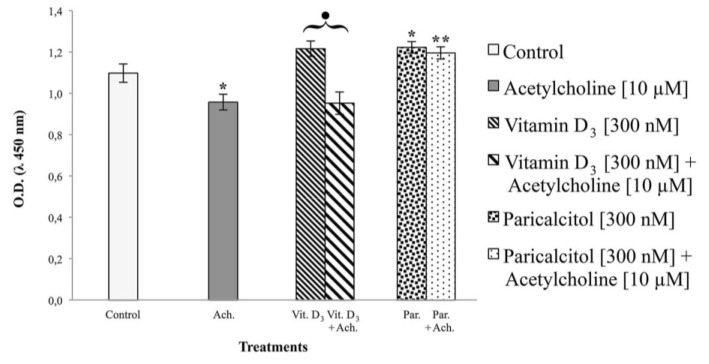
[10 μM] acetylcholine, acting on H9c2 by interaction with muscarinic M2 receptors, induces a significant decrease in mitochondrial activation in comparison to untreated cells. When [300 nM] vitamin D_3_ and [10 μM] acetylcholine are at the same time present in the medium, no changes are observed; on the contrary, when [300 nM] paricalcitol and [10 μM] acetylcholine are present in the medium, the effect of acetylcholine is abolished. Data are expressed as means ± S.E.M. *vs.* control. * Statistically significant difference (*p* < 0.05) of paricalcitol *vs.* controls (untreated cells and vehicles). ** Statistically significant difference (*p* < 0.05) of paricalcitol + acetylcholine *vs.* acetylcholine. Black dot indicates a statistically significant difference between vitamin D_3_ and vitamin D_3_ + acetylcholine.

Treatment with [10 μM] acetylcholine induces, as expected, a significant decrease in the mitochondrial activity of H9c2 whereas stimulation with [300 nM] vitamin D_3_ (or [300 nM] paricalcitol) induces a significant increase in mitochondrial activity in comparison to control, untreated cells (Figure 6).

When [10 μM] acetylcholine and [300 nM] vitamin D_3_ are simultaneously present in the culture medium, no significant changes are observed in comparison to treatment with acetylcholine alone, but when [10 μM] acetylcholine and [300 nM] paricalcitol are used (instead of vitamin D_3_) the effect of the neurotransmitter is completely counteracted.

### 3.5. Cell Calcification

**Figure 7:**
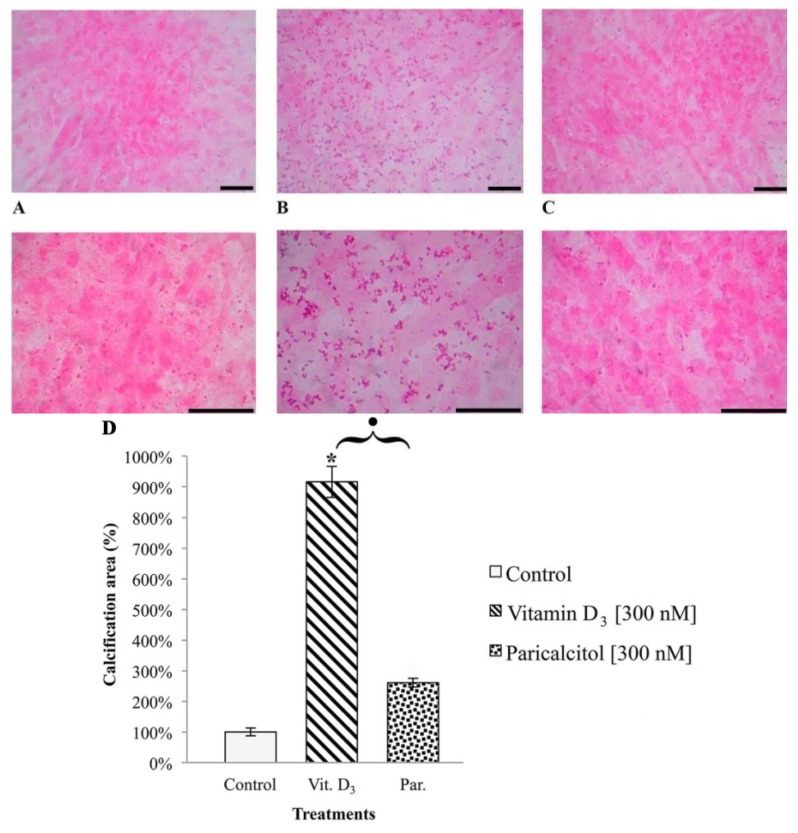
Effect of vitamin D_3_ and paricalcitol on intracellular H9c2 calcification. Panels **A**: control and vehicles treated cells. A background weak intracellular calcification is present: colored dots (dark pink) are evident into the cell cytoplasm. Panels **B**: [300 nM] vitamin D_3_ treatment induces a strong intracellular calcification in H9c2. Panels **C**: [300 nM] paricalcitol induces an intracellular calcification at the same extent of that observed in controls. Upper panels: total magnification 100×. Lower panels: total magnification 200×. Bars: 100 μm. Panel **D**: percentage of calcification area. * Statistically significant difference (*p* < 0.01) in calcification induced by vitamin D_3_ *vs.* controls (untreated cells). Black dot indicates a statistically significant difference (*p* < 0.01) in calcification induced by vitamin D_3_ *vs.* paricalcitol.

Figure 7 shows the effect of Vitamin D_3_ and Paricalcitol on intracellular calcification occurring in H9c2 cardiomyocytes. [300 nM] Vitamin D_3_ induces a significant increase in calcium incorporation in the cells in comparison to control cells (Figure 7A,B). [300 nM] Paricalcitol does not have a statistically significant effect on calcium content in H9c2 cardiomyocytes in comparison to control, untreated cells (Figure 7C).

## 4. Discussion

Recent observational studies provided support for a possible protective role for vitamin D_3_ in the CKD population [1,2]. Recent scientific evidence suggests that vitamin D_3_ may negatively regulate the renin-angiotensin system, inhibit cardiomyocyte hypertrophy and proliferation, as well as modulate and suppress the inflammatory response to blood vessel injury [18]. Cardiovascular diseases, especially atherosclerosis, have been reported to be the main causes of dialysis-related morbidity and mortality [2]. In the development of these vasculopathies, not only are traditional risk factors involved, but also CKD-related biochemical changes such as hypocalcemia, vitamin D deficiency and hyperparathyroidism, which are considered promoting factors. Previous *in vitro* studies [19] have demonstrated that endothelial cells stimulated with low Ca^2+^, high advanced glycation end products (AGEs), and parathyroid hormone (PTH), responded by increasing the expression and production of pro-inflammatory and atherosclerotic parameters such as interleukin 6 (IL6), nuclear factor kappa B (NFκB), and endothelial nitric oxide synthase (eNOS). These CKD-related biochemical changes are significantly counteracted by vitamin D_3_ treatment, which induces a decrease in the elevated IL-6 mRNA expression, positively affects the activity of NFκB, and normalizes the parameters associated with the eNOS system. 

On the other hand, other studies highlighted that an excess of vitamin D_3_ increases the risk of hypercalcemia and vascular calcification, thus worsening CV risk and reducing survival in patients with CKD [4,20]. In fact, vitamin D_3_ belongs to the category of general VDR activators, having a wide range of affinity for the components of the vitamin D_3_ system, both for the vitamin D_3_-binding protein and for the nuclear VDR. Among the VDR activators, selective activators such as Paricalcitol play a major role in controlling mineral metabolism. These selective molecules act more efficiently on parathyroid glands rather than on intestine and bone; this leads both to a lower serum calcium and phosphorus increases and to an improvement of hyperplasia of the parathyroid gland and secondary hyperparathyroidism [15]. For these reasons, selective VDR activators could provide a vitamin D-like protective efficacy without the hypercalcemic and hypophosphatemic collateral effects, thus representing a potential therapeutic tool against the cardio-renal syndrome.

From data presented in this study, it emerges that selective activators of the VDR such as Paricalcitol are not only associated with less side effects in comparison to vitamin D_3_ but they also can also act directly on cardiomyocytes inducing morphological changes, differentiation, and variations in cell ability to respond to acetylcholine. The most notable differences *in vitro* were observed at high doses; this phenomenon could be interpreted considering the peculiar mode interaction between VDR and the genes that it regulates. A differential occupancy of the receptor might in fact be responsible for the difference in the array of genes that are being regulated by VDR [21].

In fact, one of the most potentially dangerous side effects associated with long term therapy with vitamin D_3,_ *i.e.*, intracellular calcification, appears to be much less relevant when the selective activator of vitamin D_3_ axis paricalcitol is used instead. From our results it appears that vitamin D_3_ and its analog paricalcitol activate different signal transduction pathways in H9c2 cells: vitamin D_3_ induces cell proliferation associated with an increase of cAMP, whereas paricalcitol induces, at the same concentration, cell differentiation, associated with cAMP decreases. In fact, low levels of intracellular cAMP are necessary to induce muscle cell differentiation of different myogenic cell lines [16] such as H9c2. It is worth remembering that these cells derive from embryonic rat ventricle; thus, they are not yet completely differentiated into non-proliferating myocytes/myotubes and they maintain the electrophysiological and biochemical properties of both skeletal and cardiac muscle tissue [16]. Data in literature demonstrate that H9c2 cell differentiation is associated with an increase of myogenin expression, a typical skeletal muscle protein [16], and with an increase of myotrophin, a protein associated with normal cardiomyocyte development [22]. 

Treatment with high concentration of paricalcitol also induces a significant increase in cell size. Cardiomyocyte hypertrophy occurs in response to numerous agonists [23] and growth factor signaling pathways that induce hypertrophy are intimately interconnected with intracellular pathways that link changes in calcium homeostasis with the reprogramming of cardiac gene expression [24]. The calcium, calmodulin-dependent protein, phosphatase calcineurin, serves as a point of convergence of these different intracellular pathways [25] and has been shown to be necessary and sufficient for hypertrophy in response to a wide range of signals *in vivo* and *in vitro* [26]. Therefore, cardiomyocyte hypertrophy in response to paricalcitol has to be interpreted as an adaptive response that parallels and accompanies cell differentiation. Future studies will assess whether this adaptive responses will occur also in fully differentiated cardiomyocytes.

Given the recent interest in the role of vitamin D_3_ deficiency in CV system and heart functionality and integrity, we investigated the effects on heart diastolic function of the components of the vitamin D_3_ axis that are known to be associated with VDR, that are vitamin D_3_, paricalcitol, and GcMAF, and preliminary data demonstrated that the components of the vitamin D_3_ axis differently affect the diastolic function. Diastolic function has been evaluated by a rapid and non-invasive method, based on ultrasounds and measuring the interval in milliseconds between aortic closure and mitral opening; this interval of time is known as iso-volumetric relaxation time (IVRT) [27]. Physiologically, it is the time that it takes to pump free calcium out of the myocardium, to produce relaxation of the myofibrils and to allow ventricular filling, and takes all the available free energy in the heart to do so. IVRT is inversely related to the cellular free energy so the higher the IVRT is, the lower the cellular free energy is. From our data emerged that, while vitamin D_3_ increased IVRT, paricalcitol significantly decreased it, thus demonstrating a positive inotropic effects on the levels of cellular free energy. In fact, these results can be interpreted as that paricalcitol increased the cellular free energy, a novel positive feature of this analog that had not been described before. The effects of GcMAF were comparable to those obtained with paricalcitol. These results can be interpreted considering that paricalcitol and GcMAF showed similar, although not superimposable, effects at the cellular and organism level in other model systems. In fact, both compounds stimulated cAMP formation in human mononuclear cells with the highest effect on the “bb” genotype of the VDR. Both paricalcitol and GcMAF inhibited the angiogenesis induced by prostaglandin E1 in the chick embryo chorionallantoic membrane [28]. This unexpected effect of paricalcitol suggests that its advantage over vitamin D_3_ might be far more ranging than simply being non-hypercalcemic. In fact, the decrease of cellular free energy in a chronic condition is at the basis of a number of signs and symptoms that are negatively associated with the prognosis. Our observation that paricalcitol and GcMAF show an inotropic effect at variance with vitamin D_3_ opens the perspective of administering this molecule in a variety of chronic conditions where the use of vitamin D_3_, although potentially beneficial, has been discouraged by the concomitant and potentially harmful side effects of vitamin D_3_. Since the majority of the results presented in this study have been conducted *in vitro* in a rat myoblast cell line, not yet completely differentiated, the results cannot be directly extrapolated to clinical recommendations. Nevertheless, such a model system could provide novel indications that can be applied to the clinic. Thus, paricalcitol is a molecule that has been safely used for years and this study demonstrates that it shows some novel effects that could be exploited in order to maximize therapeutic effects while, at the same time, minimizing side effects. The stimulation of cardiomyocyte viability and differentiation without inducing intracellular calcification, and the positive inotropic effect in the absence of hypercalcemia, might therefore represent novel fields of application of this molecule.

## 5. Conclusions

Biochemical modifications, promoting factors and pro-inflammatory interleukins play a crucial role in the development of cardiovascular diseases in CKD patients as well as in the increase of mortality in most of the chronic diseases. Vitamin D_3_ treatment counteracts most of the CKD related modifications in cellular homeostasis and signaling decreasing the elevated IL-6 mRNA expression, positively affecting the activity of NFκB and normalizing the eNOS system. However it has been demonstrated that vitamin D_3_ long-term treatment is strongly related to vascular calcification with consequent reduction of patients’ survival. For this reason selective activators of VDR (such as paricalcitol) and factors involved in the vitamin D_3_ axis (as GcMAF) might represent new therapeutic tools both for treatment of CKD and for treatment of chronic conditions where the use of vitamin D_3_, although potentially beneficial, has been discouraged for the harmful side effects.
